# Investigating the effect of distance between the teacher and learner on the student perception of a neuroanatomical near-peer teaching programme

**DOI:** 10.1007/s00276-016-1700-3

**Published:** 2016-05-25

**Authors:** Jonny R. Stephens, Samuel Hall, Matheus Gesteira Andrade, Scott Border

**Affiliations:** Division of Medical Education, Faculty of Medicine, Centre for Learning Anatomical Sciences, University of Southampton, Mailpoint 845, South Academic Block, Tremona Road, Southampton, SO16 6YD UK

**Keywords:** Near-peer teaching, Neuroanatomy, Medical education, Social congruence, Cognitive congruence

## Abstract

**Purpose:**

Near-peer teaching (NPT) is a highly valuable resource for the education of medical undergraduates with benefits to the students, teachers themselves, and the faculty. To maximise the effectiveness of such teaching programmes, the aim of this study was to determine how the student learning experience, and underpinning social and cognitive congruencies changes as the learner–teacher distance increases.

**Methods:**

Second-year medical students at the University of Southampton participated in a series of neuroanatomy, extra-curricular revision sessions taught by the third-, fourth-, and fifth-year medical students and junior doctors. The students completed a validated questionnaire after the session rating various aspects of the teaching.

**Results:**

Although all teachers delivered sessions that we rated highly with a mean perceived gain in knowledge of 18 % amongst all students, it was found that the third- and fourth-year medical students delivered a session that was rated significantly better than the fifth-year students and junior doctors across all, but one areas of feedback.

**Conclusions:**

We believe that these findings may be explained by the diminishing social and cognitive congruencies shared between learner and teacher with increasing distance. From our results, we hypothesise that graduation is an important threshold, where there is a significant drop in congruencies between the learner and teacher, therefore, having a significant impact on the perception of the NPT session.

## Introduction

The range of teaching methods available to the medical educator is constantly increasing under the pressure to maintain teaching quality in the face of diminishing curriculum time and resources [7, 8, 30]. Near-peer teaching (NPT) is an educational method that has widely been used through informal applications, such as teaching on the wards or, more recently, through it being integrated into a formal teaching programme. In anatomy education, the practice of NPT has a long established history, which is still very much part of existing teaching approaches [1, 11, 15, 18–20, 24].

The defining characteristic of NPT is the proximity between the student and teacher both in terms of age and stage of training. The teacher, being only a short distance ahead of their students in educational training, is more able to accurately remember their own learning experiences, including the associated pitfalls of the process, which they can draw upon in the classroom [9]. Without the social barriers that may hinder academic discussion with faculty members, the students and teachers are able to engage in more open discourse regarding the learning and can easily identify any issues, where the students need extra assistance [18]. The significance of this close proximity in learning experiences was first recognised by Schmidt [27] who termed them cognitive and social congruence, respectively. Since then, the concept of congruence has been considered an important part of explaining NPT success [18, 21, 27].

The use of NPT in anatomy education has historically involved senior medical students and junior doctors assisting anatomy lecturers as demonstrators in practical sessions. Near-peer demonstrators use this experience as preparation for their future careers typically in surgery or radiology. However, NPT in undergraduate neuroanatomy has demonstrated that altering the proximity of learners and teachers in terms of educational distance has an important influence on how the teachers are perceived. Such studies identify that junior doctors tend to receive lower feedback scores for various aspects of the learning experience when compared to senior medical students [14]. One theory suggests that this variability is due to the declining strength of social and cognitive congruence [14, 21, 27]. However, as yet there is a paucity of examples in the published literature to fully delineate the dynamic nature of these congruencies along the NPT spectrum.

Given the evidence supporting the role of social and cognitive congruence in the success of the NPT learning experience, it has become increasingly important to identify when congruence is optimal between teacher and learner. When the distance increases to the point, where these elements no longer have a strong influence, it could mark the transition from NPT- to cross-level teaching. Moreover, further evidence in this area would allow more effective resource allocation and provide the best learning experience for the students.

The primary aim of this study was to determine how the student learning experience, and thus, the underpinning social and cognitive congruence, changes as the learner–teacher distance increases.

### Null hypothesis

‘There is no discernable optimal distance along the near-peer teaching spectrum in terms of students’ perceptions to their teaching in a neuroanatomy NPT context’.

## Materials and methods

Faculty endorsed optional small-group neuroanatomy revision sessions were organised at the University of Southampton for the second-year medical students. These were delivered immediately prior to their head and neck module examinations in 2012–2013 and 2013–2014. Neuroanatomy was chosen for the revision sessions, since it is recognised that students find it the most challenging (so called neurophobia), and therefore, would benefit from extra support on the subject [16, 17]. All second-year students of their respective cohorts (*n* = 245 and *n* = 247) were invited to attend the sessions through group emails, posters, and lecture announcements during the duration of the semester.

**Fig. 1:**
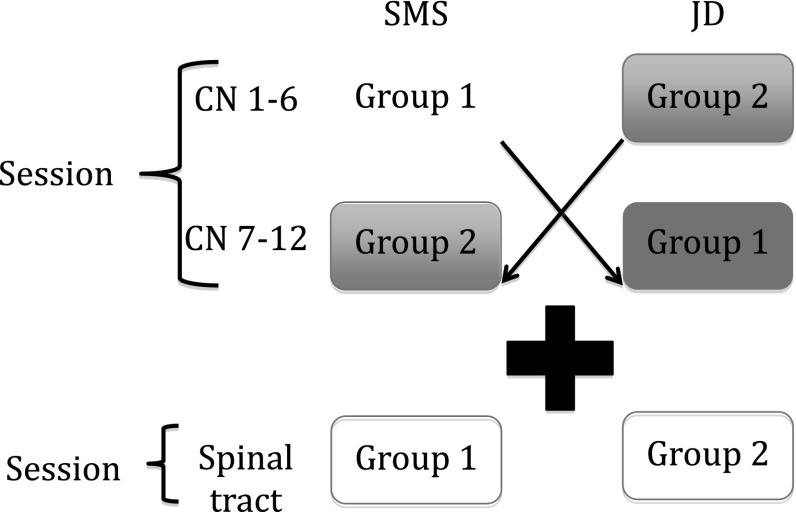
Diagram illustrating the organisation of the NPT sessions. Session 1 was divided into two 1-h-long sessions, where group 1 was taught cranial nerves 1–6 by an SMS initially and later taught cranial nerves 7–12 by a JD and vice versa for group 2. During session 2, the students were distributed evenly between the SMS and JD teachers for a 1-h-long session on the spinal tracts

Each year, the revision classes comprised three 1-h-long sessions, where the cranial nerves were split into two 1-h sessions and spinal tracts taught within 1 h. These topics were chosen based on their clinical importance and reputation, as being the most troublesome areas for students. The groups varied between 8 and 12 students depending on attendance. The sessions were taught by a series of the third- (3YMS), fourth- (4YMS), fifth (5YMS)-year medical students and junior doctors (JD) who were rotated, so that students received a different teacher type for each of the three sessions (Fig. 1). The teachers were all volunteers who showed an enthusiasm either for teaching or the subject area of neuroanatomy and taught all three sessions where logistically possible. They were selected for their enthusiasm and willingness to teach rather than their neuroanatomical knowledge. All student teachers subsequently received a certificate confirming their participation in the NPT sessions which could be used for their portfolios. The student learners completed a previously validated (Cronbach alpha score—0.83) paper-based Likert-style survey anonymously at the end of each session. The survey examined multiple perception criteria ratings of the session/teacher. These criteria were statistically determined to be part of either social or cognitive congruence and validated using the principal component analysis. Students were blinded to the academic status of their teachers and the purpose of the feedback forms.

The teachers were briefed to use standardised learning outcomes and resources which were reviewed by the Faculty’s anatomy department; however, the teachers were free to deliver their session in a format of their choice.

For a more meaningful comparison of NPT definitions within the existing published literature, the third-, fourth-, and fifth-year medical student groups were combined to give an overall senior medical student (SMS) group. This group was then compared to the junior doctors (JD).

All data were tested for the normality of distribution and the statistical analysis conducted using SPSS Ver 21. Ethical approval for this study was granted by the University of Southampton Ethics committee (Ethics ID 799).

## Results

### Student’s perceptions of the teaching

Four hundred and sixty-four feedback forms were collected from a total of six separate sessions over two academic years (2012 to 2013—255 and, 2013 to 2014—209). The sessions were well received with high ratings for all teacher types and overall session quality. (4.00–4.27/5). Students felt that their learning improved as a result of attendance, since their perceived level of knowledge rating after the session was significantly greater than it was before (74 % afterwards vs 56 % before the session—Wilcoxon Sign Ranked Test *p* < 0.0001).

### Learner perceptions of medical student and junior doctors as teachers

The overall quality of the teaching was rated highly for both the SMS and JDs at 4.26 and 4.00, respectively, albeit with the SMS scoring significantly higher than their JD counterparts (*p* = 0.005) (Table 1). In addition, five of the remaining eight feedback criteria were rated significantly higher for the SMS teachers with two non-significant criteria approaching the *p* < 0.05 level of significance. The five significant differences were: relevance of content (*p* < 0.0001), ability to solve weaknesses (*p* = 0.001), delivery of session (0.006), use of time (*p* < 0.0001), and enjoyment of the session (*p* = 0.007).

**Table 1:** Feedback criteria assessed on the questionnaire with associated ratings for, and comparisons between, the SMS and JD

	SMS mean average rating (max score 5)	JD mean average rating (max score 5)	*p* value
Overall	4.26	4.00	0.005
Relevance of content	4.59	4.24	<0.0001
Explanations	4.26	4.10	0.205
Ability to solve weaknesses	3.90	3.53	0.001
Delivery	4.20	3.92	0.006
Use of time	4.19	3.77	<0.0001
Approachability of teacher	4.60	4.41	0.067
Confidence about exams	3.49	3.29	0.069
Enjoyment	3.96	3.65	0.007

Those rating that showed statistically significant differences between groups are highlighted in blue

### Learner perceptions of third-, fourth-, and fifth-year medical students and junior doctors as teachers

When comparing all four-teacher types using Mann–Whitney *U* all areas of feedback, except the students becoming more confident about their exams and the quality of the explanations, were significantly different (Table 2). The 464 feedback forms were attributed to the following teacher type: 3YMS-192, 4YMS-176, 5YMS-36, and JD-60.

**Table 2:** Feedback criteria assessed on the questionnaire with associated ratings for, and comparisons between, each teacher type

	3YMS mean average rating (max score 5)	4YMS mean average rating (max score 5)	5YMS mean average rating (max score 5)	JD mean average rating (max score 5)	*P* value comparing all groups
Overall	4.27	4.26	4.03	4.00	0.005
Perceived knowledge increase (%)	0.99 (20 %)	0.89 (18 %)	0.66 (13 %)	0.80 (16 %)	0.006
Relevance of content	4.54	4.64	4.25	4.24	<0.0001
Explanations	4.25	4.28	4.03	4.10	0.200
Ability to solve weaknesses	3.87	3.92	3.74	3.53	0.011
Delivery	4.18	4.25	3.94	3.92	0.006
Use of time	4.17	4.21	4.00	3.77	0.002
Approachability of teacher	4.65	4.55	4.25	4.41	0.011
Confidence about exams	3.45	3.51	3.47	3.29	0.307
Enjoyment	3.96	3.94	3.66	3.65	0.012

Those rating that showed statistically significant differences between groups are highlighted in blue

Indeed, the best overall rating and best perceived knowledge increase were found for the 3YMS teachers, closely followed by the 4YMS. Post-hoc analysis revealed no significant differences between 3YMS with 4YMS groups. Similarly, when comparing the 5YMS with JDs, there were no significant differences to report. However, when comparing the 4YMS and 5YMS, there were five feedback items, where the 5YMS received significantly worse scores than the 4YMS. These areas were: perceived knowledge gain (*p* = 0.011), delivery of session (*p* = 0.017), enjoyment of the session (*p* = 0.047), relevance of content (*p* = 0.002), and approachability of the teacher (*p* = 0.033). Therefore, in summary, the 3YMS and 4YMS were rated significantly higher than 5YMS and JDs in multiple areas of feedback.

## Discussion

Near-peer teaching is a concept that has been practiced for many years within anatomy education [1, 11, 15, 18–20, 24]. The countless benefits of this approach for the students, teachers, and anatomy departments are already well documented [5, 10, 22, 24, 25]. In more recent times, the drive towards modern professionalism led curriculums has led to a decline in the amount of timetabled sessions available for basic science subjects like anatomy compounded by a reduced number of qualified anatomy educators [19, 29]. Therefore, NPT programmes can be viewed as one of a variety of possible solutions to maintain standards of knowledge and confidence in the subject. With this in mind, it becomes increasingly important to scrutinise the benefits of NPT, particularly if they are to become formally incorporated as part of curriculum delivery, as it has done at The University of Southampton.

The benefits of NPT to the teacher have been previously well-described by Reyes- Hernandez [24] and Bulte [4] with benefits to the faculty explored in a study by Duran [8]. Our investigations have focused more on the benefits to the student learners with aims and objectives similar to that of Evans and Cuffe [11] only with a stronger emphasis on the educational distance between tutors and students. Our sessions garnered a high overall rating for the quality of the session as well as consistently high ratings for all areas of feedback across all teacher types, highlighting the value of both the SMS and JDs acting as near-peer teachers. Furthermore, in the current study, students taught by all four-teacher types reported an increase in their perceived knowledge as a result of attending the session (Table 2). This would suggest that this type of teaching is an effective means to impart knowledge, and that all student teachers successfully fulfilled the role of tutor. This finding, in turn, adds further support to the growing weight of published evidence supporting the successful use of medical students as teachers in the education of their colleagues [1, 8, 10, 19, 22, 23, 26]. In addition, it not only argues in favour for the application of NPT initiatives within medical education, but also provides evidence to suggest that NPT revision programmes can be fully integrated into an established anatomy curriculum.

Previous work in this field suggests that increasing the educational distance between the student and teacher could have a detrimental effect on how the teacher is perceived by the student [14]. This could, in part, be explained by social and cognitive congruencies as well as the increase in professional identity associated with graduation. By identifying when the influence of social and cognitive congruence changes, it may be possible to not only better define NPT, but also reveal other potential factors, in addition to distance, which contribute to the success of NPT.

Our current study is in agreement with Hall [14] who reported that SMS significantly outperform JDs in multiple areas of NPT feedback. We found that 3YMS and 4YMS were rated significantly higher than 5YMS and JDs in multiple areas of the survey (Table 2). However, when assessing the feedback gained about the 3YMS and 4YMS, there was no significant difference between the two teacher types in any area of the evaluation. This was also the case when comparing 5YMS and JDs and might suggest a possible ‘drop-off’ in social and cognitive congruence along the NPT spectrum.

Specifically, two important areas of social congruence, approachability of the teacher and enjoyment of the session, were rated significantly higher for 3YMS and 4YMS compared to the 5YMS and JDs. This leads us to speculate that the teachers may no longer be able to be a ‘student among the students’; something frequently cited as a defining feature of social congruence [21, 27].

While it may be true that near-peer facilitators have less formal experience as teachers, their greatest asset may be their ability to bring a student’s perspective to their role [8, 9]. The student, to a large extent, relies on the expertise and experience of a teacher to select the most relevant knowledge and teach it in the most appropriate way [6]. Students reported that the content taught by the third- and fourth-year medical students during the sessions was significantly more relevant to their needs and delivered in a better way than the sessions by a 5YMS or a JD (Table 2). Junior doctor led NPT in prescribing and examination skills was rated as very relevant by the final-year students [29]. The material selected by JDs for NPT may be suitable for clinical subjects to senior students, but becomes less relevant for the second-year students, if they focus on the clinical aspects of the topics, as opposed to the core neuroanatomy principles needed for the second-year exams. This was a theme mirrored by anecdotal comments given by the students.

There are multiple factors involved as to what makes an effective teacher. One key factor is the motivation of the teacher themselves which will come along with cognitive congruence, but inversely with an increased learner- teacher distance. Teacher motivation will subsequently impact on other areas of how a teacher can be assessed, including preparedness of the session, teacher enjoyment of the session, and so forth. These are areas that could be assessed in future sessions as well as to incorporate teacher feedback to better understand what effect the motivation of the teacher has on how they are perceived by their students.

**Fig. 2:**
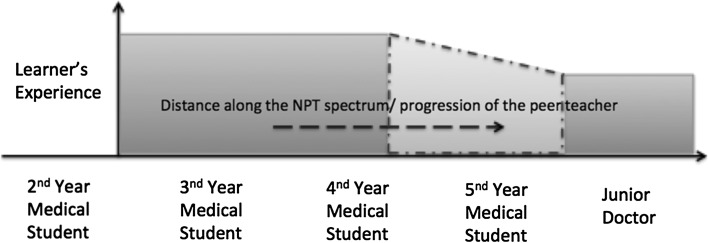
Schematic of the NPT spectrum, depicting how the ‘learners experience’ of a NPT session varies with increasing distance between the learner and the student. Note the area in *red*, the so called ‘transition zone’ before the proposed threshold of graduation

The results of the current investigation strengthen the theory that graduation appears to be an important threshold in terms of how a teacher is perceived by their students [14]. We have measured the learners experience based on the overall rating of the sessions and depicted the hypothesis (Fig. 2) that this is reduced over what we called a transitional zone period, which is represented around graduation time. Graduation is an academic, personal, and social transition, suggesting that it impacts on how a teacher is perceived [2, 12]. On entering the final year, the student is expected to behave less like a student and more like a practicing doctor. With this change comes the altered mindset, including the realisation of future job stresses, responsibility and the increased requirement for autonomy. Although this training equips the final-year medical students with the skills and attitudes required for working life as a doctor, the drawback is that they share less cognitive and social congruencies with their junior medical student colleagues, and this may impact on their success as a teacher at undergraduate level [3].

Interestingly, one of the only areas of feedback that did not show significant differences across all teacher types was how confident the teaching made the students feel about their forthcoming examinations; this was also the area of feedback that was universally rated the lowest. Asking medical students to rate their confidence about examinations may be insensitive to differences in teacher type because of student’s natural fear of examinations and failure to progress. It is well known that students are inherently poor at judging their own ability [13, 28].

Given the fact that NPT has a variety of conflicting definitions, these results may allow further refinement of the NPT definition [4, 11, 18, 26]. Bulte et al. [4] state that the students and their teachers must be ‘at the same level of the medical education spectrum’ for it to be truly near-peer teaching. From our experience, we propose that distance between the teacher and learner, irrespective of any hierarchical boundaries, should be considered in future enhancements of the NPT definition.

### Limitations to the study

Although we believe this study has many merits, it is important to acknowledge its limitations. It has yet to be seen whether this ‘drop-off’ is a manifestation of a 2-year gap between learner and teacher, or is in fact, a change in outlook that occurs specifically between the fourth- and fifth-year medical students. Further research is required to ascertain whether this two-year gap is transferrable to any point along the teaching spectrum and if these results are generalisable to other subjects aside from neuroanatomy.

As a control measure for experience, we did not allow teachers who had taught twice previously on our NPT programme to teach again; however, we did not control the amount of other teaching experience they may have had which may act as a cofounding variable. It is also important to note that questionnaire items may be open to a degree of subjectivity from the user; however, this again utilises the student’s perceptions, a key element to this study. It is acknowledged that there were greater number of 3YMS and 4YMS teachers compared to 5YMS and JD teachers. This is due to the demands of the final-year curriculum and work commitments of these teachers thereby reducing their availability.

Finally, despite appropriately showing differences between the groups using non-parametric statistics, the Likert rating scale of 1–5 may not offer the best discrimination for the comparison of the groups, and thus, consideration for increasing the rating scale will be taken for further studies,

### Recommendations

Considering the popularity and success of NPT sessions at Southampton, we continue to strongly recommend the use of near-peer teachers in undergraduate medical curriculums to enhance subjects like anatomy. From our own observations, we tentatively propose that a distance of two educational years between learner and teacher provides a greater probability for a positive learning experience. We propose that lower levels of clinical and academic experience are outweighed by higher levels of social and cognitive congruencies when it comes to the teaching of one’s peers in an informal setting.

It has recently been suggested that the most important attribute of a successful neuroanatomy teacher is simply the degree of dedication and enthusiasm [6]. From our experience, we have found that NPTs can provide these qualities to usefully supplement neuroanatomy education alongside that delivered by trained and experienced experts. Although formal training has been recommended for near-peer teachers to produce better and sustainable outcomes [6, 8, 23, 24], we believe that a lack of training should not stop senior medical students acting as near-peer teachers in a formal context, particularly, if they have the support of academics in their faculty to build a sustainable programme. An effective alternative to formal training is a strong collaboration between the NPTs and senior teachers. In this manner, they would be able to provide support the NPTs prepare for their teaching sessions, but also reflect on the sessions to instigate change in areas that need it.

## Conclusion

Educational distance along the NPT spectrum has been emphasised as an important factor within the application of NPT. Specifically, progression from 3rd year to 4th year had a little influence on the way, the teachers are perceived by learners. However, entering into the final year of medical school and the proximity to graduation seems to be important in terms of the differences between student-ratings of the teachers. We are in agreement with many previous investigations in concluding that changes in social and cognitive congruence are at least partly responsible for this. Not withstanding the value of an enthusiastic and pedagogical “classical” anatomy teacher, NPT has continued to show promise of being a useful tool in maintaining a satisfactory anatomical level of medical students given an ever growing pressure on curriculum time, diminishing resources, etc. If anatomy education begins to rely more on NPT programmes in the future; investigations, such as this may be an important step towards understanding how the student experience can be maximised.

## References

[1] Anstey LM, Michels A, Szyums J, Law W, Ho MHE, Yeung RTT, Chow N (2013). Reflections as near-peer facilitators of an inquiry project for undergraduate anatomy: successes and challenges from a term of trial-and-error. Anat Sci Edu.

[2] Bleakley A (2002). Pre-registration house officers and ward-based learning: a ‘new apprenticeship’ model. Med Educ.

[3] Brennan N, Corrigan O, Allard J, Archer J, Barnes R, Bleakley A, Collett T, de Bere SR (2010). The transition from medical student to junior doctor: today’s experiences of Tomorrow’s Doctors. Med Educ.

[4] Bulte C, Betts A, Garner K, Durning S (2007) Student teaching: views of student near-peer teachers and learners. Med Teach 29(6):583–590 10.1080/01421590701583824 17922356

[5] Burgess A, McGregor D, Mellis C (2014). Medical students as peer tutors: a systematic review. BMC Med Educ.

[6] Chang BS, Molnar Z (2015). Practical neuroanatomy teaching in the 21st Century. Ann Neurol. doi:10.1002/ana.24405. Accessed online 20 May 10.1002/ana.24405 25810129

[7] Drake RL, McBridle JM, Lachman N, Pawlina W (2009). Medical education in the anatomical sciences: the winds of change continue to blow. Anat Sci Edu.

[8] Duran CE, Bahena EN, Rodriguez Mde L, Baca GJ, Uresti AS, Elizondo- Omana RE, Lopez SG (2012). Near-peer teaching in an anatomy course with a low faculty-to-student ratio. Anat Sci Edu.

[9] Durning SJ, Ten Cate OTJ (2007). Peer teaching in medical education. Med Teach.

[10] Erie AJ, Starkman SJ, Wojciech P, Lachman N (2013). Developing medical students as teachers: an anatomy-based student-as-teacher program with emphasis on core teaching competencies. Anat Sci Edu.

[11] Evans DJ, Cuffe T (2009). Near-peer teaching in anatomy: an approach for deeper learning. Anat Sci Edu.

[12] Finlay SE, Fawzy M (2001). Becoming a doctor. Med Humanities.

[13] Finn GM, Sawdon M (2010) Factors influencing student’s ability to self and peer assess performance. Association for Medical Education in Europe (AMEE), Conference 2010 Glasgow, UK, 4–8 Sep 2010

[14] Hall S, Stephens J, Andrade T, Davids J, Powell M, Border S (2014). Perceptions of junior doctors and undergraduate medical students as anatomy teachers: investigating distance along the near-peer teaching spectrum. Anat Sci Edu.

[15] Houwink AP, Kurup AN, Kollars JP, Frai Kollars CA, Carmichael SW, Pawlina W (2004). Help of third-year medical students decreased first year medical students’ negative psychological reactions on the first day of gross anatomy dissection. Clin Anat.

[16] Jozefowiez R (1994). Neurophobia: the fear of neurology among medical students. Arch Neurol.

[17] Kramer B, Soley J (2002). Medical student perception of problem topics in anatomy. East Afr Med J.

[18] Lockspeiser TM, O’Sullivan P, Teherani A, Muller J (2008). Understanding the experience of being taught by peers: the value of social and cognitive congruence. Adv Health Sci Educ Theory Pract.

[19] Lockwood AM, Roberts AM (2007). The anatomy demonstrator of the future: an examination of the role of the medically-qualified anatomy demonstrator in the context of tomorrow’s doctors and modernizing medical careers. Clin Anat.

[20] McCuskey RS, Carmichael SW, Kirch DG (2005). The importance of anatomy in health professions education and the shortage of qualified educators. Acad Med.

[21] Moust JHC (1993) De rol van tutoren in probleemgestuurd onderwijis. Contrasten tussen student-en docentutoren. [On the role of tutors in problem based learning: contrasting student-guided with staff-guided tutorials] [doctoral thesis] Maastricht, The Netherlands: University press

[22] Nelson AJ, Nelson SV, Am Linn, Raw LE, Kildea HB, Tonkin AL (2013). Tomorrow’s educators…today? Implementing near-peer teaching for medical students. Med Teach.

[23] Noel GPJC (2014). Why more anatomy departments should embrace near peer teaching with inter-professional demonstrators. Austin J Anat.

[24] Reyes-Hernandez CG, Carmona Pulido JM, De la Garza Chapa RI, Vazquez RPS, Briones RDA, Banda PMP, Silva EEV, Baca GJ, Castro OG, Omana REE, Lopez SG (2015). Near-peer teaching strategy in a large human anatomy course: perceptions of near-peer instructors. Anat Sci Edu.

[25] Robinson Z, Hazelgrove-Planel E, Edwards Z, Siassakos D (2010). AMEE guide supplements: peer-assisted learning: a planning and implementation framework. Guide supplement 30.7—practical application. Med Teach.

[26] Rodrigues J, Sengupta A, Mitchell A, Kane C, Maxwell S, Cameron H, Ross M, Ford M (2009). The Southeast Scotland Foundation Doctor Teaching Programme—is near-peer teaching feasible, efficacious and sustainable on a regional scale?. Med Teach.

[27] Schmidt HG, Moust JH (1995). What makes a tutor effective? A structural-equations modeling approach to learning in problem-based curricula. Acad Med.

[28] Stephens J, Hall S, Andrade MG, Border S (2014) Can medical students accurately self-assess their own knowledge gain in a near-peer teaching setting? Association of Medical Education Annual Scientific Meeting, Brighton: 2014 July 16–18

[29] Van Mameren H (2004). Source of future teachers of anatomy. Anat Rec B New Anat.

[30] Wilson FC (2009). Graduate medical education: issues and options.

